# Is the “Theory-theory” of Understanding a Mistake? An Interactive Perspective on Knowledge and Explanation

**DOI:** 10.1007/s42113-026-00298-4

**Published:** 2026-04-24

**Authors:** Simon Myers, Nick Chater

**Affiliations:** Behavioural Science Group, Warwick Business School, Coventry, UK

## Abstract

Shiffrin, Stigler, and Keil worry that science frequently falls victim to an illusion of understanding. We take their argument a step further, arguing that psychological scientists often fundamentally mischaracterize human understanding as rooted in internally represented *theories* of any kind. We propose an alternative, interactive notion of understanding.

Shiffrin, Stigler and Keil (SSK) make a persuasive case that scientists often labour under an illusion of understanding: a sense that they possess deeper, more coherent explanations than they really do. We suggest that this illusion reflects a more fundamental mistake. It is not merely that our internal theories are shallow, but that understanding does not consist in the possession of theory-like structures at all. We tend to conceive of understanding as organised around general, internally represented principles from which particular judgments are derived. In this commentary, we argue that this “theory-theory” of understanding (e.g., Carey, 2009; Carey & Spelke, 1994; Gopnik et al., 1999; Gopnik & Wellman, 1992; Perner, 1991; Wellman, 1990,1) is misguided. Human cognition is not primarily a matter of applying internal theories, but a capacity for flexible, context-sensitive improvisation in interaction with the world.

When we think about learning physics, it is natural to imagine that we are internalising something like the theories of electromagnetism or thermodynamics. Likewise, when learning to add, multiply and so on, it is tempting to think that we are acquiring something like an approximation to the closure of the implications of the axioms of arithmetic (Piaget, 1952). Indeed, beyond the sciences, similar assumptions pervade philosophy, where conceptual analysis (Jackson, 1998) presupposes that, beneath a superficial layer of jumble and confusion, we possess stable and coherent concepts that can be organised, regimented, and made precise (see Machery, 2009; Williamson, 2007; Wilson, 2006; Wittgenstein, 1953 for attacks on this viewpoint). Rational choice theory in economics (e.g., Becker, 1976) similarly presumes that, underlying the apparent volatility and inconsistency of human judgment and decision-making, there exists a stable and consistent core of beliefs—each with an associated probability—and desires, that is, representations of outcomes each associated with some subjective utility (Fishburn, 1970; Savage, 1954/2012; Von Neumann & Morgenstern, 1944/2007). These domains, too, are replete with paradoxes and contradictions, as well as with vast areas in which our moral intuitions or decision-making apparatus are ill-specified, vague, inapplicable or otherwise unhelpfully silent (Haidt, 2001; Hauser et al., 2007; Tversky & Kahneman, 1982). Our judgments are volatile, our concepts fragmentary, and our intuitions frequently in tension. Why might this be? We suggest that this is not a superficial failure to approximate an underlying theory, but a reflection of the fact that everyday human performance does not depend on around internal representations of coherent, theory-like representations at all (Chater, 2018).

Of course, it is widely recognised that our understanding is only approximate, in part because we routinely make mistakes—such as calculation errors in arithmetic—and because we are constrained by limits of computational tractability (Gigerenzer & Selten, 2002; Lieder & Griffiths, 2020; Simon, 1955), and, in the extreme, by formal results such as Gödel’s theorem (Gödel, 1931/1992). Still, it is easy to imagine that what we have internalized is, at least approximately, an abstract theory. However, on our view, understanding consists not in the application of abstract theories, but rather in the capacity for contextual improvisation—responding appropriately within a specific situation. Such understanding typically unfolds through interaction: with another person, with a problem, or with the external world itself (e.g., Clark & Chalmers, 1998; Chater, 2018; Dennett, 1987; Dreyfus & Dreyfus, 1986). If we reflect on our intuitions about what it means for a person to understand a topic better than we do, we typically mean that they can survive sustained cross-questioning and navigate novel cases. Crucially, this cross-questioning may involve direct engagement with the natural world. In many domains—chemistry, animal behaviour, medicine—such understanding is most convincingly demonstrated not in words alone, but in the laboratory, the field, or the clinic.

SSK rightly emphasizes that understanding is a matter of degree. Even apparently simple questions can open into indefinitely deep explanatory chains, and any explanation can itself become the target of further “why” questions (Aristotle, 1994; Lombrozo, 2006). This threatens an infinite regress. In practice, however, such regresses are not as troubling as one might fear. What matters is not whether an agent can provide ultimate explanations, but whether they can answer questions, make predictions, or act in ways that are satisfactory to achieve the local goals at hand (Myers & Chater, 2024). Understanding is inherently pragmatic and comparative: we ask whether one person understands better than another, or whether someone understands well enough to meet the current demands. In this sense, understanding is fundamentally a matter of local satisficing rather than global optimization.

SSK’s discussion ranges not only over the cognition of individual scientists but over scientific understanding itself. Here it is crucial to distinguish between the cognitive resources of human thinkers and the formal structures they construct. Scientific theories—mathematical models, symbolic systems, computational simulations—are public artifacts. They support forms of deduction, precision, and internal coherence that far exceed what any individual mind can survey or internalise. Such systems acquire a kind of autonomy from human intelligence: at least within parts of the theory, they have a life of their own (i.e., the *theory* has predictions, not just its creators). We agree with SSK that the predictive success of a theory does not, by itself, license strong claims about its truth or completeness; but it does license provisional preferences among competing models.

It is worth noting, though, that many of the apparent paradoxes discussed by SSK with regards to modelling and regression do not arise from inconsistencies in the mathematical formalisation itself, but from attempts to read causal structure directly out of statistical relationships, without first fixing the causal questions at issue or ensuring that the data are capable of discriminating among the theories in question (Pearl, 2009; Pearl et al., 2016; Spirtes et al., 2000). We suggest that one way to minimize problems of this type is to generate candidate *causal* theories first and then evaluate them by comparing their predictive performance on new data.

One virtue of formal theories is that they can often generate precise point-predictions rather than merely qualitative or directional expectations. This precision enables stricter and more discriminating tests. But it comes at a cost. Highly formalised models typically apply only within narrow domains and therefore tend to be less, rather than more, general. Their power lies not in breadth but in the possibility of deductive exploration: by running a mathematical or computational model, we can derive consequences that far outrun unaided human intuition (Bechtel & Abrahamsen, 2005; Winsberg, 2019). In this sense, scientific understanding is often scaffolded by external cognitive tools—mathematics, simulations, and physical computers—rather than implemented within the head (Clark, 2010; Clark & Chalmers, 1998).

The intuitive “paradoxes” discussed by SSK that surround regression are instructive here. They show how easily informal interpretation outruns what narrow formal models actually warrant. But the reason why these are *intuitive* paradoxes and not real paradoxes is precisely the mismatch between the formal theories external to the mind and their necessarily shallow, fragmentary, and context-bound representations within the mind. Even where formal methods do seem applicable, our intuitions remain prone to paradox. Indeed, there are innumerable paradoxes across logic, mathematics, probability theory, physics, and semantics (Chater, 2025; Sorensen, 2003). Our intuitions are riddled with vast gaps as well as internal contradictions - exactly as one would expect if cognition is not organised around internally coherent theories. Nor should their formal coherence be projected back onto human cognition. The fact that a symbolic system is unified and precise does not imply that it could be or should be mentally represented as such.

Indeed, the most precise formal theories tend to apply only within narrow domains. Physics and physical chemistry provide striking examples, but even here crystalline islands of mathematical tractability are surrounded by vast territories—biology, geology, climate science, and certainly psychology, and the social sciences—in which explanation is pluralistic, approximate, and heavily dependent on modelling choices and background assumptions.

Much of what we call scientific understanding consists not in the deployment of unified laws, but in the skilful navigation of messy, heterogeneous bodies of evidence. The ubiquity of paradox, vagueness, and apparent contradiction in human thought is therefore not an anomaly. Our intuitions are fragmentary, context-bound, and often mutually inconsistent. Logic, probability, semantics, and even mathematics teem with puzzles that resist unification. From the present perspective, this is exactly what we should expect if cognition is not organised around internally coherent theories, but around the capacity to cope, case by case, with an open-ended range of demands.

The history of artificial intelligence provides a useful parallel. Early symbolic approaches treated intelligence as the manipulation of explicit, internally represented rules and theories. Their limited and brittle successes revealed how difficult it is to specify, in advance, the structures that human competence appears to require (Brooks, 1991; Dreyfus, 1992; Haugeland, 1989). The fact that our intuitions are both ill-specified and mutually inconsistent has made it seems impossible to formalise many fragments of human knowledge, and effectively ground the symbolic AI programme to a halt (Brooks, 1991; Dreyfus, 1992; Haugeland, 1989). So much of it relies on metaphorical and analogical leaps, grounded in perceived similarities rather than in precise systems of logical rules. By contrast, contemporary machine-learning systems achieve strikingly flexible performance without operating over explicit, unified theories (Bommasani et al., 2021; Brown et al., 2020; Bubeck et al., 2023). They succeed by developing vast repertoires of context-sensitive dispositions shaped by experience, enabling them to meet the demands of particular tasks—whether communicative exchanges, the sequencing of moves in games such as chess or Go (Silver et al., 2016, 2018), performance in Atari environments (Mnih et al., 2015), or the construction and interpretation of images (Ramesh et al., 2022; Rombach et al., 2022)—through often astonishingly flexible generalisations over often very large numbers of experiences rather than reasoning from general explanatory models^2^. Whatever their ultimate limitations, these systems demonstrate that effective cognition need not depend on the possession of internally coherent theories. They offer a mechanistic illustration of how shallow, piecemeal resources can nonetheless support powerful forms of interaction with complex environments.

Why then are we so tempted to think of understanding as theory possession? We suggest that our linguistic and symbolic abilities are misleading us (Brooks, 1991; Rumelhart & McClelland, 1986). Because we can articulate rules, definitions, and explanations, and because we can externalise them in durable symbolic forms, it is natural to suppose that such structures must also underwrite cognition itself. But verbal and mathematical systems are best seen as tools we use to build and interrogate external theories, not as transparent windows onto the organisation of the mind. Only a tiny fragment of human knowledge has been codified in formal terms and decades of effort in symbolic AI strongly suggest that most of what underlies everyday competence is not readily captured in unified rule-based systems.

SSK’s illustrations of the illusion of explanatory depth are therefore of broad significance. We suggest, however, that this illusion is rooted in a deeper misconception: the assumption that understanding consists in the internal possession of ever deeper explanations. Rather, we wish to say that understanding is not something the mind *has*, but something the mind *does*. In most areas of knowledge, we suspect, we may have to accept the inevitable shallowness, and often contradictory, nature of understanding, in both humans and AIs. Yet this very shallowness enables us, nonetheless, to successfully interact with a world far too complex for the creation of theory.

## Data Availability

No datasets were generated or analysed during the current study.

## Footnotes

1 We use ‘theory-theory’ here not to donate specific theories in theory-of-mind research (e.g. Gopnik & Wellman, 1994) but to denote the broader assumption that understanding consists in the internal possession of coherent, theory-like structures.

2 While modern AI systems are remarkably capable without internal symbolic structure, it is important to note that perhaps the jury is still out on whether this can be done away with entirely. Prominent voices in the field such as Gary Marcus and Yan LeCun remain convinced that, for AI systems to meet human capability, they still need to have explicitly built-in world models (Garrido et al., 2024; LeCun, 2022; Marcus, 2020; Mollo & Millière, 2023).
